# Impact of Deposition of the (TiB_x_/TiSi_y_C_z_) x3 Multilayer on M2 HSS on the Cutting Force Components and Temperature Generated in the Machined Area during the Milling of 316L Steel

**DOI:** 10.3390/ma15030746

**Published:** 2022-01-19

**Authors:** Agnieszka Twardowska, Łukasz Ślusarczyk, Marcin Kowalski

**Affiliations:** 1 Institute of Technology, Pedagogical University, ul. Podchorazych 2, 30-084 Krakow, Poland; marcin.kowalski@up.krakow.pl; 2 Faculty of Mechanical Engineering, Cracow University of Technology, al. Jana Pawla II 37, 31-155 Krakow, Poland; lukasz.slusarczyk@pk.edu.pl

**Keywords:** multilayer coatings, TiB_2_, M2 HSS cutting tools, PLD, milling force components, temperature during dry milling, wear

## Abstract

High-speed steel (HSS) tools account for 20 percent of the cutting tools materials’ global market. This is due to both their significant toughness and resistance to cracking, compared to cemented carbides. Covering steel tools with hard coatings clearly improves their mechanical properties, wear resistance, and significantly increases their durability. Physical vapor deposition methods are preferred for coating metal substrates, as they allow low temperature deposition. The most widely deposited coating materials are carbides, nitrides, and borides. They are combined with softer ones in the multilayer structure to promote increased resistance to cracking and delamination in comparison to monolayered structures. In this paper, the M2 steel end mills were coated by (TiB_x_/TiSi_y_C_z_) x3 multilayer by the pulsed laser deposition method. Coated and uncoated tools were tested in the cylindrical down milling of AISI 316L steel. Components of the cutting force and temperature generated in the machined area during dry milling were measured under two variants of operating conditions: V1 and V2. Tool wear mechanism was examined using scanning electron microscopy (SEM), accompanied by EDS analysis of worn areas. It was found that milling with higher speed (variant V2) is accompanied by lower cutting force components and a lower temperature generated in cutting area. The presence of the coating allowed lower cutting forces and temperature in the case of variant V1. The temperature measured during milling did not exceed 200 °C. The SEM observation of the edges of cutting tools indicated that the main mechanism of wear for both types of tools was abrasion. The built-up edge formation was observed in the case of tools tested at the V1 cutting parameters variant. It was assumed that it was the reason for higher cutting forces measured during milling according to this variant. The chemical composition of built-up edges was different for coated and uncoated tools. Tribo-chemical reactions were responsible for the reduction of the cutting force and temperature components observed during milling with a coated tool at V1 variant. Boron and titanium were the elements of the coating that enabled the tribo-oxidation reactions thanks to which friction was reduced. Our results show that this beneficial effect occurs with (TiB_x_/TiSi_y_C_z_) x3 coated tools, but can easily be lost with inadequately selected cutting parameters.

## 1. Introduction

Machining is one of the most important methods of shaping products, and the most commonly represented is milling with a market share of machining technologies estimated at 39%, followed by turning at 30% [1,2]. Modern machining technologies, such as high-speed machining, high-performance machining, and dry machining, create extreme working conditions for cutting tools [3]. The development of machining technologies requires the development of tool materials which can meet such working conditions [2,3]. The problems of high temperatures and cutting forces in the cutting zone that adversely affect tool life and wear is very important [4,5,6]. The experimental methods for temperature and cutting force determination in the cutting zone are described in the literature [7,8]. Cutting tools materials used nowadays in different machining technologies are mainly cemented carbides—with 53% of all cutting materials used, high speed steels (20%), and ceramic, PcBN, and PCD (together 19%) [1,2]. It is clear that despite the development of new tool materials, the HHS tools are still in use, as they are less expensive than cemented carbides and they have better bending strength and fracture toughness. The main methods used to improve the properties of HSS tools are the use of powder metallurgy methods for their production or/and coating [9,10,11]. Hard and wear-resistant coatings significantly improve the properties of coated HSS tools, allow working with higher cutting parameters, and maintain low machining costs related to the costs of the cutting tool. In addition, unlike other tool materials, worn HSS tools can be regenerated/re-coated [12], which is economically and ecologically beneficial. According to [1,2] more than 60% of cutting tools are coated, mainly by chemical vapor deposition (CVD) method. Among end mills, 74% are coated and the main method of coating them is physical vapor deposition (PVD). The deposition method and its parameters strongly influence the microstructure and properties of coatings. The level of internal stresses that arise during the deposition is an important factor for the tool life. In the case of TiB_2_ coatings, the internal stresses built up is high, up to −10 GPa [13], and usually it is accompanied by high levels of hardness, but their compressive nature is unfavorable. In the presence of external loads, highly stressed coatings show strong tendencies to the formation of cracks and delamination from substrates. Therefore, intensive research has been carried out to develop the PVD methods and determine deposition parameters that ensure the production of coatings with the lowest possible level of internal stress. One of the developed directions is to use energetic ions. It was demonstrated that by magneton sputtering of a TiB_2_ target, it is possible to obtain TiB_2_ coatings of a relatively low stress of −500 MPa [14]. While in the case of the dual-beam ion beam assisted deposition (DB IBAD) method [15], it is possible to obtain TiB_x_ coatings of intrinsic stress of the tensile nature (<1 GPa).

TiB_2_ is very attractive for wear resistant applications because of both high hardness and resistance to wear. Unfortunately, monolayer TiB_2_ coatings are susceptible to cracking and delamination from metal surfaces. To use its advantages, such as a high melting point, thermal stability, good thermal and electric conductivities [16], TiB_2_ is combined with softer phases capable of absorbing the excess energy of cracking, for example Ti [17], C and MoS_2_ [18], TiC [19], and TiSi_y_C_z_ [20]. In this case, we also used TiSi_y_C_z_ as interlayer, in a multilayer coating which was deposited (using the PLD method) alternately with TiB_x_ layer to form a coating on the M2 steel substrate. A novelty in our research was a prototype HSS tool with a new type of coating, with respect to the type of layers and their arrangement. It is a new concept, as the most widely researched nitride coatings on HSS tools are based on TiN, TiAlN, AlTiN, TiCrN, and TiCN phases [9,10,11,12]. In our (TiB_x_/TiSi_y_C_z_) x3 multilayer, the good characteristics of the TiB_x_ layer (hardness, wear resistance, thermal stability) are combined with the ability to absorb the energy of damage to the TiSi_y_C_z_ layer. We applied an adhesive layer of amorphous Ti, which allowed us to obtain high adhesion of the multilayer coating to steel substrate, which is usually difficult to achieve due to significant differences in the properties of metals and ceramics (e.g., linear thermal expansion, the lack of mutual wettability). The microstructure and mechanical and friction-wear properties of produced coating-substrate systems are presented and discussed in detail in as-deposited state and after short post-deposition annealing in our previous papers [21,22]. Our multilayer coatings offer a significantly improved performance in comparison to single- or bi- layered coatings. The aim of the current paper is to investigate the performance of the (TiB_x_/TiSi_y_C_z_) x3 coated M2 steel tools under real working conditions and to compare it with the performance of uncoated tools tested in the same conditions. These studies are intended to verify the promising test results obtained previously in friction-wear tests, showing an anti-wear effect of the coating by increasing the wear resistance and reducing the coefficient of friction (with steel as a counterpart). Thus, the current research is aimed at assessing the suitability of the tools as potential cutting tools for machining stainless steel. To reach the goal, commercially available M2 HSS end mills were used as substrates, and, after coating deposition, they were used in dry cylindrical down milling of AISI 316L steel in two variants of milling parameters. During the milling in both variants the components of cutting force and temperature generated in the cutting zone were measured and analyzed.

## 2. Materials and Methods

### 2.1. Targets and Substrate Materials

Amorphous (Ti_x_/TiSi_y_C_z_) x3 coatings were deposited on AISI M2 high-speed steel (HS 6-5-2) end mills by the PLD method without additional substrate heating. Table 1 shows the characteristics of materials (targets and substrates) used in our experiments.

**Figure 1:**
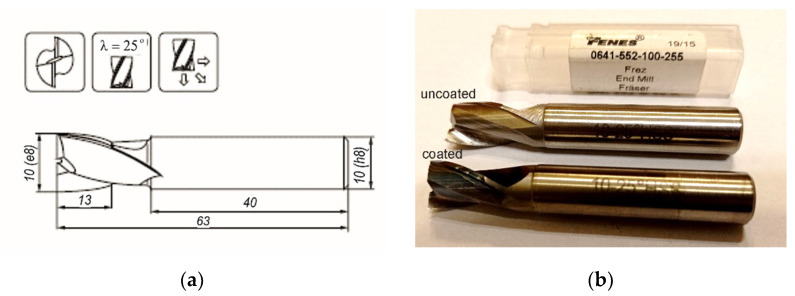
FENES DIN 327-B K (0641-552-100-255) end mill (**a**) geometry, (**b**) photographic image of M2 HSS end mills, uncoated and coated on the working part.

As substrates, we used commercial two-blade FENES (FENES S.A., Siedlce, Poland) DIN 327-B K 0641-552-100-255 (10 mm) end mills of the geometry shown in Figure 1. Before the coating deposition, substrates were pre-degreased in acetone, then ultrasonically cleaned in distilled water, degreased in pure isopropanol, and dried in air. The coating was produced by subsequent deposition of TiB_x_ and TiSi_y_C_z_ layers. The TiB_2_ target (purity 99.8 wt.%, Goodfellow, Darmstadt, Germany) and the Ti_3_SiC_2_ target (purity 97 wt.%, IOS Institute of Advanced Manufacturing Technologies, Krakow, Poland), were used, each in a form of a disc of 50 mm in diameter and ~5 mm in thickness. Prior to deposition, the targets were mechanically ground and polished on one side using diamond grinding and polishing suspensions (9 ÷ 1 µm). After each step, the sample was ultrasonically cleaned in a distilled water bath, then degreased in pure isopropanol and dried (in air). The pre-ablation procedure was carried out to reveal a clean area of targets, free from mechanical treatment residues and adsorbed gas contaminations.

### 2.2. Pulsed Laser Deposition Parameters

An Nd: YAG laser (Powerlite Precision II 9010 DLS, Continuum, Paris, France) was applied, operating at λ = 266 nm, E = 102 mJ, energy density (fluency) ρ = 2.03 J/cm^2^, with repetition rate of 10 Hz and pulse duration of 3–6 ns. The pulsed laser deposition process was performed in Neocera chamber, at 10^−4^ Pa vacuum. The geometry of PLD was vertical with the substrate placed parallel above the target. The target–substrate distance was ~70 mm. Targets were placed on separate rotating tables of the carousel system [21]. The Nd: YAG laser beam was focused on the target’s surface, with a 45° angle of incidence. For a single layer, the deposition time and deposition rate were set to 15 min and ~4 nm/min, respectively. These deposition parameters make it possible to obtain a single layer of ~65 nm thickness and the coating thickness of ~400 nm.

### 2.3. Milling Test Parameters

**Figure 2:**
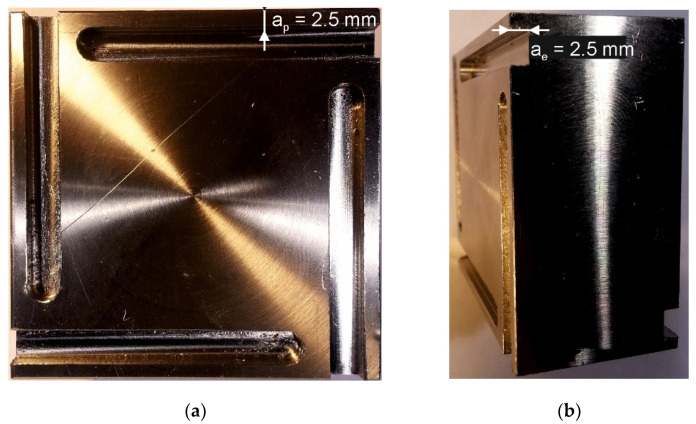
The AISI 316L sample: (**a**) side view, with a_p_ [mm], (**b**) front view, with a_e_ [mm] parameters selected.

The milling tests were carried out at room temperature (35–40% humidity), without the use of liquid coolants, under two variants of operating parameters of cylindrical down milling (V1, V2), as shown in Table 2. Figure 2 shows the AISI 316L steel sample; (a) side view with a_p_ [mm] and (b) front view, with ae [mm] parameter selected. Operating parameters were selected for the milling of 316L steel with M2 HSS end mills by trial tests, carried out with the use of uncoated tools. The signal representing the cutting force components was recorded with 1000 Hz frequency. Each trial to mill the workpiece was performed twice, for coated and uncoated tools. A new tool was used for each trial. The average surface roughness of 316L samples after milling was determined by analysis of images taken by a confocal microscope Olympus LEXT in the XYZ scan mode of areas of 128 µm × 128 µm.

### 2.4. The Measuring Station for Recording Components of Milling Force Components and the Temperature in the Milling Zone

**Figure 3:**
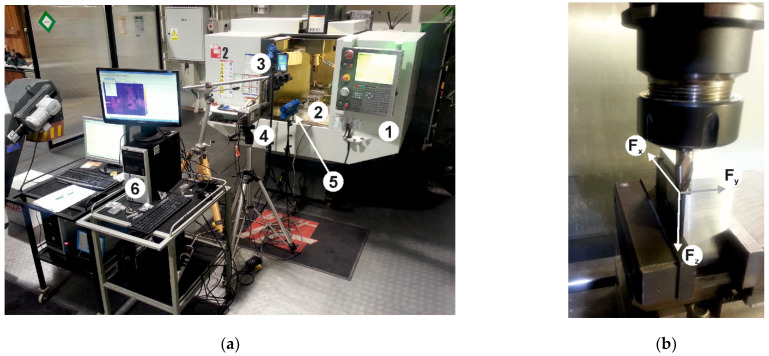
(**a**) The set used as the measuring station: 1. HAAS Mini Mill 2; 2. piezoelectric force gauge KISTLER type 9257B with tool holder (The Kistler Group, Winterthur, Switzerland); 3. FLIR SC620 thermal imaging camera connected to a PC computer via FIREWire cable working with ThermaCAM Researcher Pro 2.9 software (FLIR Company, Wilsonville, OR, USA); 4. PHANTOM V5.2 high-speed changeable frame camera (Vision Research, Inc., Wayne, NJ, USA) with a fixed lens NIKKOR AF 200 mm focus lens, connected to computer via ethernet cable compatible with Cine 663 software 5. Lighting: spot (cold light), Dedocool (Coolt3 model), 6. PC computer with software enabling the acquisition of measurement results (cutting force: DynoWare, quick change image capture—Cine 663 app, thermogram registration: ThermaCam Researcher Pro 2.9 app), (**b**) Components of milling force measured during cylindrical milling.

**Figure 4:**
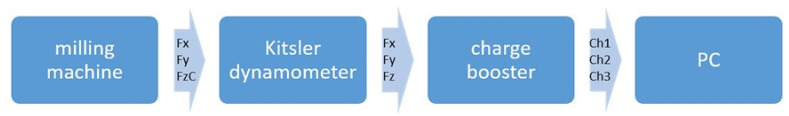
Diagram of the measuring track for recording the components of the cutting force.

Figure 3a shows the measuring station for recording the milling force components, temperature generated in the milling zone, and for recording high-frame changeable images. Figure 3b shows the spatial orientation of the components of milling force measured during the milling. The diagram of the measuring track for recording the components of the cutting force is shown in Figure 4.

The high-frame changeable camera was used to observe the milling process with respect to chip formation and its evacuation from the cutting area. It was placed in front of the cutting zone and captured at 1800 fps at 1024 × 512 px. The color depth was 12 bits, and the exposure time was set at 550 µs.

### 2.5. Microstructure Examinations

The scanning electron microscope (SEM) JEOL JSM-6610LV (JEOL Ltd., Tokyo, Japan) was used to examine the wear mode of end mills’ blades after the milling tests. The SEM observations were accompanied by the chemical composition analysis of worn areas by the energy-dispersive X-ray spectroscopy (EDS) method, using the X-Max detector (Oxford Instruments, Abingdon, UK), equipped with Aztec 2.1 software.

## 3. Results and Discussion

### 3.1. Components of Cutting Force and Torque

**Figure 5:**
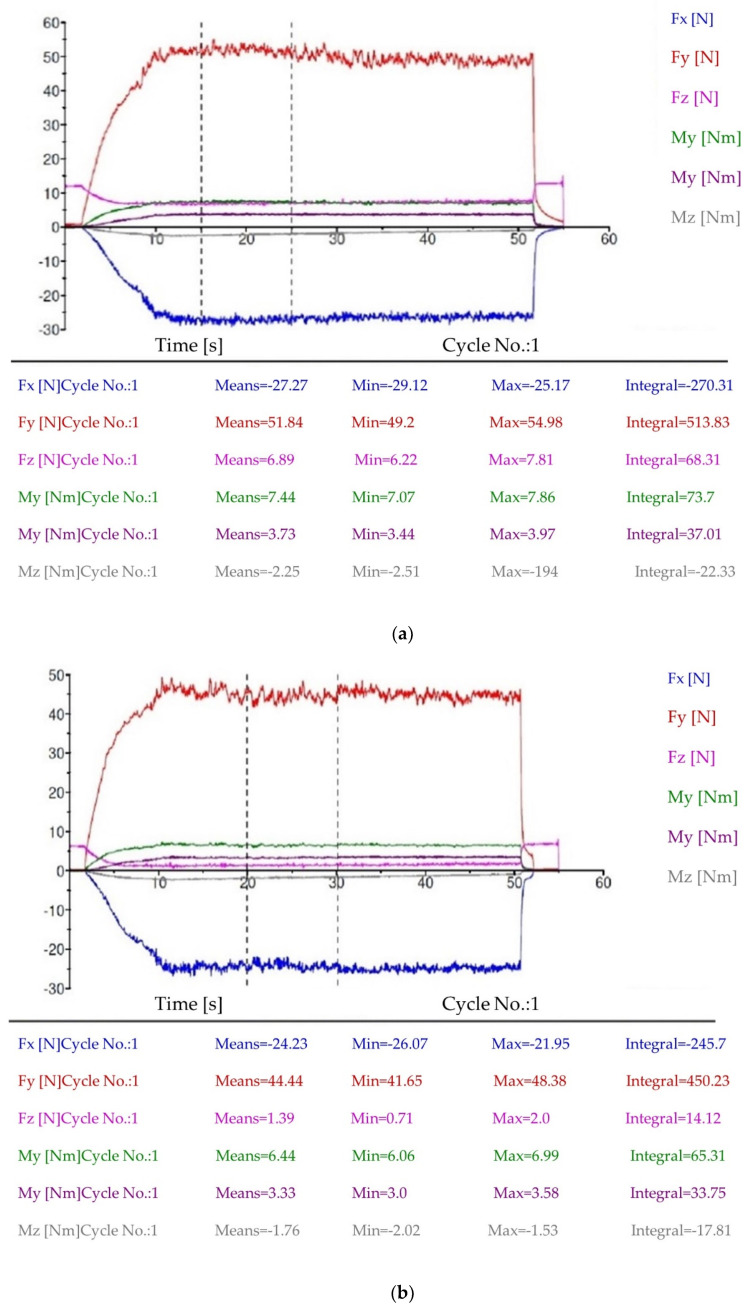
Components of cutting force (F_x_, F_y_, F_z_) and torque (M_x_, M_y_, M_z_), recorded during milling of AISI 316L steel with (**a**) uncoated and (**b**) coated M2 HSS tools, machining variant V1.

Figure 5 presents changes in the values of the components of cutting force F (N) and cutting torque M (Nm) as a function of time, registered during the milling of AISI 316L steel with uncoated and coated M2 HSS tools at V1 operating parameters. Milling at the V1 operating parameters variant with the use of multilayer coated tools was accompanied by visibly lower values of the cutting force components (Figure 5a) throughout the whole test period, compared to machining with the uncoated tools (Figure 5b). The cutting torque components determined for the coated tools were also lower. Since the measurement of the components of forces and cutting torque concerns the cylindrical milling, the main forces were components F_x_ and F_y_ of the force (Figure 3b).

**Figure 6:**
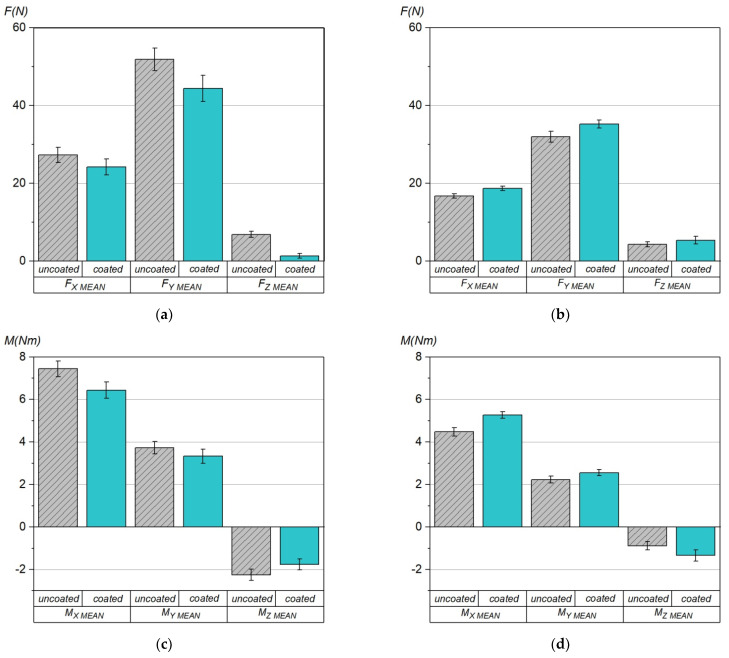
Average values of components of cutting force (F_x_, F_y_, F_z_) and cutting torque (M_x_, M_y_, M_z_), recorded during milling of AISI 316L steel with uncoated and coated HSS tools, at operating parameters: (**a**,**c**) V1; (**b**,**d**) V2.

Figure 6 shows the mean values of the components of cutting force and cutting torque, recorded during milling tests at V1 and V2 operating parameters. It can be clearly seen that milling with higher cutting parameters V2 takes place at the reduced cutting force and torque. The effect of reducing cutting force when increasing the cutting speed is often observed while maintaining other cutting parameters [24], and is used to increase the machining efficiency. Unfortunately, unlike in the milling according to the variant V1, the measured components of force and torque are higher for coated tools.

### 3.2. Temperature in the Milling Area

The following calibration parameters for the FLIR SC620 thermal imaging camera were adopted: camera temperature range: 0–500 °C, object emissivity: ε = 0.6, object distance: 1 m, ambient temperature: 20 °C, recording frequency: 30 Hz. As an f = 38 mm fixed-focus lens was used in the thermovision studies, the minimum distance that guaranteed the image sharpness was about 100 cm. The measured area included elements of various surface emissivity, e.g., workpiece, chip formed during the machining process, and a fragment of the cutter holder. After the tests and preliminary studies, the averaged emissivity value of 0.68 was used for the analyzed area. The average maximum temperature and the maximum temperature values registered during milling at V1 and V2 parameters are given in Table 3.

**Figure 7:**
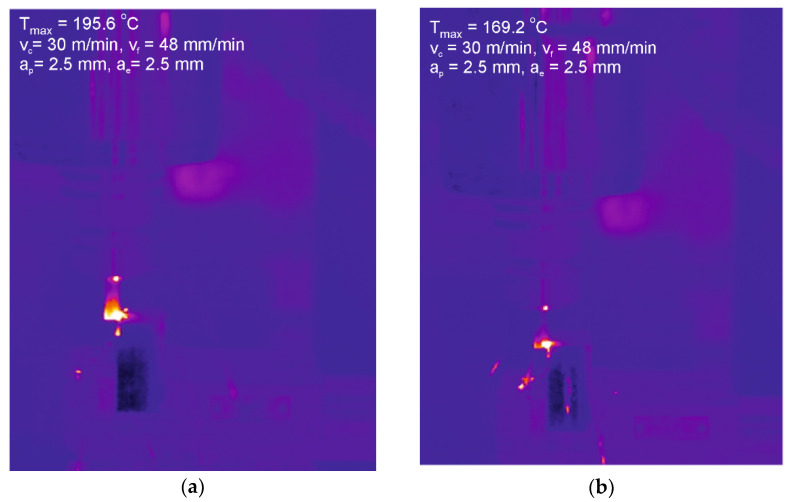

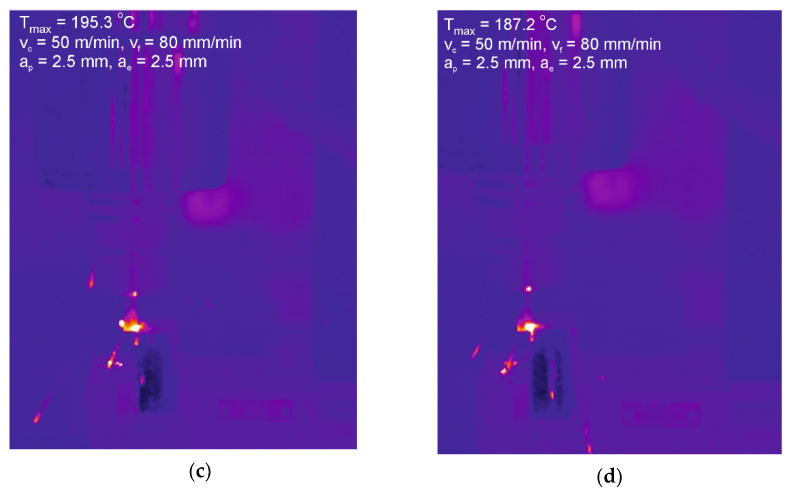
Thermograms showing the maximum temperature registered during the milling of AISI 316L steel, (**a**,**b**) V1 operating parameters, (**c**,**d**) V2 operating parameters, (**a**,**c**) uncoated M2 HSS, (**b**,**d**) coated M2 HSS.

The mean temperature values measured in the cutting area were several °C lower in the case of the coated tools compared to the uncoated, regardless of the machining variant. The maximum temperatures registered during milling fluctuated considerably. Figure 7 shows images registered during milling of AISI 316L steel, with uncoated mill and coated HSS tools at V1 operating parameters. In both cases (V1, V2), the highest temperature (195.6 °C) was measured when milling with an uncoated M2 HSS tool.

### 3.3. Wear Mechanism- SEM Observations

**Figure 8:**
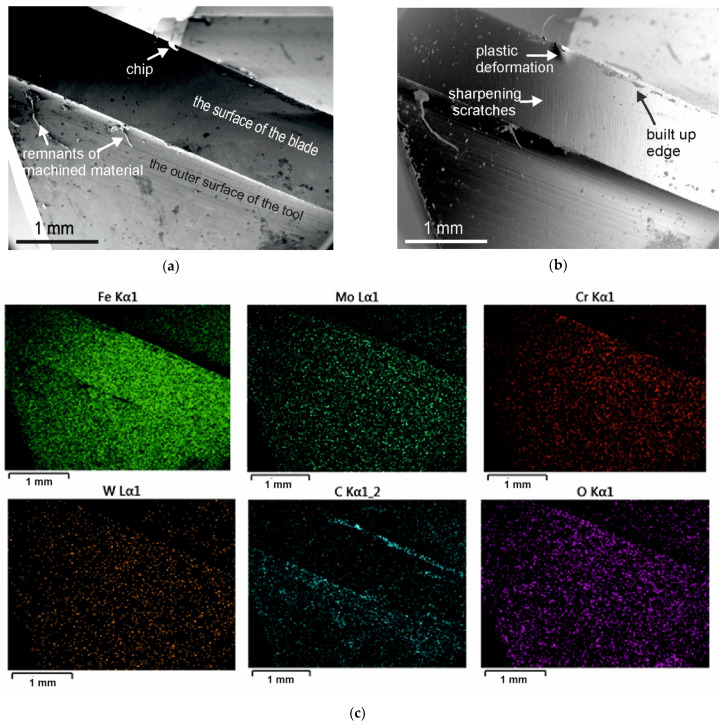
SEM images of the M2 steel tool after milling of AISI 316L steel (**a**) SEI—secondary electron image; (**b**) BET—backscattered electron image in topo mode, (**c**) EDS maps for Fe, Mo, Cr, W, O, and C.

**Figure 9:**
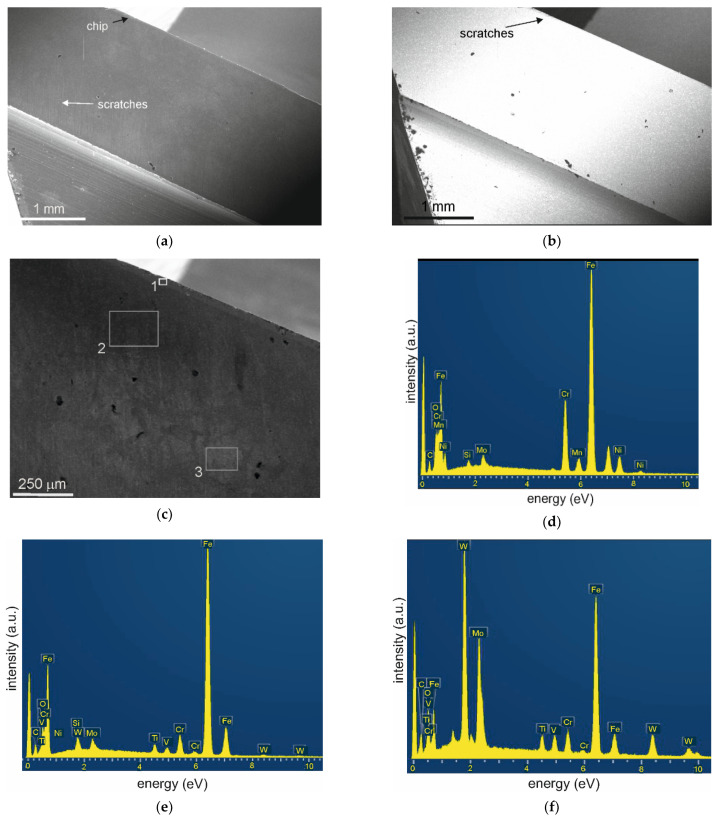
Microstructure and chemical composition analysis of the M2 steel tool blade coated with the (TiB_x_/TiSi_y_C_z_) x3 multilayer after milling of AISI 316L steel V1 variant (**a**) SEI general view, (**b**) BEC- general view, (**c**) SEI magnified view of the worn blade with built-up edge, along with marked area for EDS analysis, (**d**) EDS spectrum 1 of the built-up edge material, (**e**) spectrum 2, (**f**) spectrum 3.

The scanning electron microscopy images of cutting edge of AISI M2 steel end mills, uncoated and coated with (TiB_x_/TiSi_y_C_z_) x3 multilayer, are shown in Figure 8 and Figure 9, respectively. The SEM observations of the surfaces of blades after the milling showed differences in the manner of their wear. The blades of uncoated cutters were worn mainly by abrasion. Scratches visible on the surfaces of the blades (Figure 8) running at an angle of ~60 degrees to the edge of the blade are traces of sharpening the tools by grinding. The depth of the scratches was clearly reduced during the cutting in the middle area of the blade to the cutting edge. Locally, at the edge of the uncoated tools, some signs of plastic deformation were observed in the vicinity of the chipping (Figure 8b) and nicks. Traces of plastic deformation prove that the yield point of the tool material has been exceeded in these areas. This type of damage was not observed on the blades of coated tools. Coated tools suffered less abrasive wear. As we have shown in the indentation tests and ball-on-disc [21] tests, the hardness and wear resistance of the multilayer coated M2 HSS substrate is significantly increased in comparison to the uncoated one. The edges of the coated blades were also damaged by spallation, but no cracks were observed on the surfaces of the coated blades. In the case of uncoated tools, the EDS showed increased carbon content at the edge area (Figure 8c). On the outside and inside surface of coated tools’ blades, working at V1 operating parameters, some areas similar to solidified droplets were visible (Figure 8b). The SEM/EDS analyses of these areas showed that they were remnants of the cut material rubbed onto the blades. A greater build-up occurred on the tools which were used to mill at a lower speed (V1 variant of milling parameters). The built-up edges could be the cause of the higher measured values of the components of force and cutting torque at lower speeds of cutting [26].

The build-up observed on coated tools was only on their edges, and it was in the form of a continuous layer. However, on uncoated blades, it also appeared on the side surface of the blade, near the edge. The built-up edge layer on coated tools contained nickel (Figure 9d) coming from the 316L steel, (M2 HSS does not contain this element, Table 1), as well as O and elements from the coating (Ti, Si).

Since no cooling lubricants were used, the liquid phase could only arise as a result of tribo-chemical reactions of the coating elements (Ti, B, and Si) with oxygen from the environment. Increased wear resistance of titanium-containing materials is connected with the tribologically induced formation of the sub-stoichiometric titanium oxides [27]. In Mo containing coatings, MoO_3_ is formed, providing a lubricating surface due to its low shear interface [28]. Similar tribo-oxidation products are reported for V, B, and N containing materials. The oxide formation on the worn surface which could act as a lubricating layer was found to significantly affect the friction characteristic of SiC–TiB_2_ [29]. In our case, the tribofilm or wear debris were difficult to analyze, because they were very thin and dispersed. Based on the EDS analysis results [21], products of tribo-chemical reactions of the coated M2 sample tested in friction contact with 100Cr6 steel contained mainly Ti, B (coating), and O, but also traces of W and V (M2 steel substrate). On the basis of current and previous research, we found that these oxides contribute to the reduction of friction, but only under certain operating conditions. They behave like solid lubricants and soften with increasing temperature but can also turn into a liquid that lubricates the surfaces and removes the heat from the contact area [30]. Products of tribo-chemical reactions are responsible for reducing the values of the cutting force components at V1 milling, but at higher cutting speeds (V2) this effect disappears. The formation of tribofilm based on easy-to-shear oxides at a worn surface can result in good lubrication but is often dependent on temperature [31]. In some cases, at elevated temperatures, good lubrication characteristics are lost by complete oxidation of the wear track, resulting in intensive abrasion and high friction. In our experiments, the temperature in the cutting area is essentially the same for both variants, so we believe that during the milling at higher operating parameters (V2), these tribo-oxidation products did not arise in sufficient amounts. During the milling, the contact time of the blade with the workpiece material is interrupted and decreases as the cutting speed increases. At higher operating parameters (V2), the cutting force components were slightly reduced. A lower cutting force means less friction in the contact area, and less tribo-chemical reaction products. Only stable and adequate amounts of oxide layer on the coating surface could provide self-lubricating characteristics [28].

### 3.4. Chip Formation and Evacuation from Cutting Area

**Figure 10:**
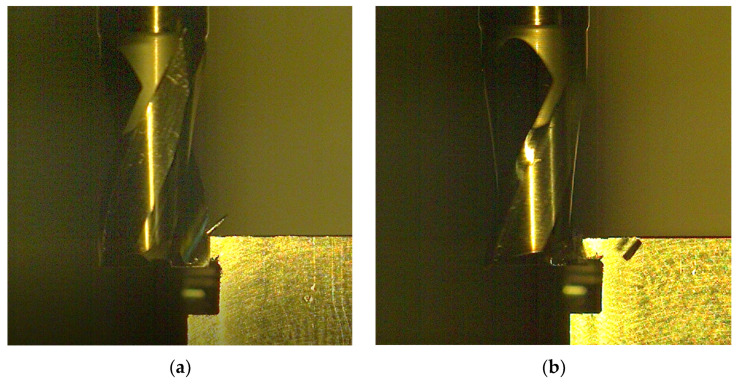

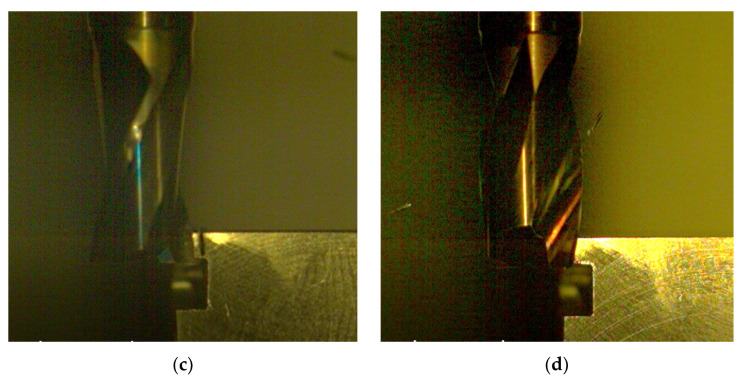
High speed camera images, recorded during the milling of AISI 316L steel in V1 (**a**,**c**) and V2 variant of operation parameters; (**a**,**b**) uncoated, M2 HSS (**c**,**d**) coated M2 HSS tool.

Figure 10 shows selected images taken from video sequences recorded during the milling of a 316L HSS sample with a coated (a) and uncoated tool (b) at operating parameters V2. Analysis of the videos revealed that the course of milling process was similar for both types of tools and variants of the milling process parameters. There were no visible differences in the chip formation. Chips were short, easily broken, and quickly evacuated from the cutting area. The roughness of the machined surfaces was at the same level ea. Ra 1.6, regardless of the tool type.

## 4. Summary

The M2 steel end mills coated with (TiB_x_/TiSi_y_C_z_) x3 multilayer by the pulsed laser deposition method were tested during the cylindrical down milling of AISI 316L steel. The components of cutting force and torque, as well as temperature generated during dry milling, were measured under two variants of operating conditions. It was found that milling at a higher speed (variant V2) is accompanied by lower cutting force components and a lower temperature generated in the cutting area. The likely cause of higher cutting forces with lower cutting data was a build-up on the blades. This is an effect often encountered in machining [26]. Our results showed that milling with coated tools reduces the milling force components and generates lower temperatures in the machined area, but only at lower operating parameters among the selected ones. On the other hand, at higher operating parameters, milling with coated tools is associated with increased cutting force components, in comparison to the uncoated tools, at a slightly higher average temperature but still lower than for the uncoated tools. The wear mechanisms of coated and uncoated tools were mainly abrasive. Locally, plastic deformation was also observed at the uncoated tool’s edges, near the chip. The wear was less intensive in the case of coated tools, which is related to their higher hardness and wear resistance [21]. The EDS analysis of blades after the milling of 316L steel indicated differences in chemical composition of built up materials present on the edges of coated and uncoated tools. In case of coated blades, elements of the 316L steel and coating (Ti and Si) were present in the tribofilm. It was assumed that the tribo-chemical reaction products were the main reason for the observed reduction of the cutting force components and temperature during the milling with a coated tool. This favorable effect was lost with increased cutting operation parameters. It was assumed that in order to maintain these attractive properties the (TiB_x_/TiSi_y_C_z_) x3 coated tools require appropriately selected cutting parameters. The analysis carried out in this study shows that the prototype tools made of M2 steel with the (TiB_x_/TiSi_y_C_z_) x3 multilayer coating may contribute to the increase of machining efficiency at specific cutting parameters. The machining processes, with their use, may be easier and less energy consuming due to the lower cutting force. Additionally, a lower temperature in the cutting area causes less heating of the tool material and the workpiece, which is beneficial. However, this should be confirmed in other machining processes, e.g., turning or drilling. This is the subject of our further research.

## Figures and Tables

**Table 1:** Characteristics of targets and substrate materials.

Item	Composition [% wt.]	Density [g/cm^3^]	Thermal Conductivity [Wm^−1^K^−1^]	Thermal Expansion ×10^−6^ [K^−1^]	Hardness	Young Modulus [GPa]
TiB_2_ target	99 (TiB_2_)	4.45	24–26 [16]	3.7–6	25 GPa	430
Ti_3_SiC_2_ target	97 (Ti_3_SiC_2_), 1 (TiC_x_), 2 (TiSi_2_)	4.42	32–37 [23]	8.6–9.7	4 GPa	320
AISI M2 steel (hardened)	Fe/C 0.9, W6, Co5, Cr4, Mo5, V2	8.13	41 [24]	10–12.6	97 HRB, 62 HRC	210
AISI 316L steel	Fe/C 0.03, Cr18, Ni12, Mo2.5, Mn2, Si1	8.00	15 [25]	16-18	80 HRB	200

**Table 2:** Variants of operating parameters of milling of AISI 316L steel.

Item	v_c_ [m/min]	v_f_ [mm/min]	a_p_ [mm]	a_e_ [mm]	l [mm]
V1	30	48	2.5	2.5	35
V2	50	80	2.5	2.5	35

**Table 3:** Mean maximum and maximum temperatures registered during the milling of AISI 316L steel sample with coated and uncoated M2 tools.

Operating Parameters	Tool	Mean T_max_ [°C]	T_max_ [°C]
V1	uncoated M2	135.2 ± 27.1	195.6 ± 3.8
	coated M2	118.3 ± 25.0	169.2 ± 3.4
V2	uncoated M2	144.9 ± 22.3	195.3 ± 3.9
	coated M2	131.0 ± 22.1	187.2 ± 3.6

## Data Availability

Not applicable.
